# Pre-earthquake non-epidemic Vibrio cholerae in Haiti

**DOI:** 10.3855/jidc.4524

**Published:** 2014-01-15

**Authors:** Jie Liu, Christopher Winstead-Derlega, Eric Houpt, Rebecca Heidkamp, Jean Pape, Rebecca Dillingham

**Affiliations:** 1 Division of Infectious Diseases and International Health, University of Virginia, Charlottesville, VA, USA; 2 Division of Nutritional Sciences, Cornell University, Ithaca, NY, USA; 3 Groupe Haïtien d’Etude du Sarcome de Kaposi et des Infections Opportunistes, Port-au-Prince, Haiti; 4 Center for Global Health, Weill Cornell Medical College, New York, NY, USA

**Keywords:** Haiti, microbiology, *V. cholerae*, epidemic

## Abstract

**Introduction:**

To our knowledge, there was no record of *Vibrio cholerae* in Haiti until the 2010 post earthquake outbreak.

**Methodology:**

This study describes the analysis of 301 stool samples from 117 infants in Port-au-Prince, Haiti, who participated in a pediatric nutrition study between July 2008 and October 2009.

**Results:**

Nine samples were identified positive with both SYBR Green and Taqman-MGB probe based molecular assays targeting *V. cholerae* hlyA and toxR, respectively (Ct = 33 – 40), but none were O1 or O139.

**Conclusions:**

Our results from multiple molecular assays demonstrate the presence of non-O1/O139 *V. cholerae* DNA in stools collected from nine asymptomatic Haitian infants two years prior to the 2010 earthquake.

## Introduction

Despite the presence of epidemic cholera in the Caribbean throughout the 1800 and 1900s, Haiti was unaffected by cholera from 1960 until several months after the devastating January 12^th^ 2010 earthquake. The cholera epidemic has killed thousands of people, and extensive efforts have focused on the etiology of this outbreak [1,2]. A recent article showed that two distinct populations of *V. cholerae* were detected in Haiti early in the epidemic and proposed that non-O1/O139 strains likely existed prior to the earthquake based on comparative genomic analysis [3]. However, all the samples tested were collected after the earthquake. Direct evidence of presence of *V. cholerae* in clinical or environmental samples prior to the earthquake would be valuable.

## Methodology

### Specimens

As part of a pediatric nutrition study presented elsewhere, we preserved 301 stool samples from 117 infants in Port-au-Prince, Haiti from July 2008 to October 2009 [4]. Surveillance stool samples were collected from study participants at 6, 9, 12, and 18 months of life. Samples were also collected from a post-intervention control group at nine months of life only. Samples were not specifically sought during episodes of diarrheal illness. Samples were aliquoted, stored at −20°C, and shipped to the University of Virginia.

### Nucleic acid extraction and amplification

DNA was extracted using the QIAamp DNA stool Mini Kit (Qiagen Inc., Valencia, USA). The protocol was modified to include bead beating with 0.15 mm garnet beads (MO-BIO Laboratories, Inc, Carlsbad, USA) for 2 minutes followed by boiling for 5 minutes before extraction. The samples were tested with PCR assays listed in Table 1.

## Results and discussion

We first screened the samples with SYBR Green and Taqman-MGB probe based molecular assays targeting *Vibrio cholerae* hlyA and toxR, respectively (Table 1), two widely used gene targets for detection of *Vibrio cholerae*. A stringent definition of positivity, i.e. positive for both targets was applied to rule out potential non-specific amplification with fecal samples and stochastic detection at lower limit of detection.

Among 301 stool samples, nine were identified as positive (hlyA Ct = 35.6 ± 2.4; toxR Ct = 36.8 ± 2.2). This finding was confirmed by amplicon sequencing. Two samples each were from stools collected at ages 6, 9, and 18 months, and three samples were from stools collected at 12 months.

We were unable to culture *V. cholerae* on Thiosulfate-citrate-bile salts-sucrose agar directly or after enrichment in alkaline peptone water, most likely due to the age of the samples, storage conditions, multiple freeze-thaw cycles, etc. Therefore further characterization was continued with DNA directly extracted from stool using the published assays in Table 1, to differentiate O1, O139 and non O1/O139, or to determine the Variable-Number Tandem Repeat (VNTR), or to test for various virulent genes. None of the samples were identified as O1 or O139 with the published assays [7]. Similar to reported genotypic data describing non O1/O139 strains [3], virulence factors, including ctxA, ctxB, zot, rtxA, rtxC, were not detectable in our samples. We further attempted to determine the multilocus Variable-Number Tandem Repeat (VNTR), a useful tool for tracking the origin of *V. choleare* [11,12]. Five loci (VC0147, VC0436/437, VC1650, VCA0171, VCA0283) were amplified and sequenced, however only one (VC0147) yielded interpretable sequence data for two samples (5 and 6 repeats, respectively) which would likely lead to their categorization as environmental non O1/O139 [3].

Amplicon contamination was ruled out, as we confirmed positivity with a separate toxR assay. In addition, *Vibrio cholerae* strains that had been handled in the laboratory were all ctxB-positive.

Due to the incapability of generating isolates from these samples and potential non-specific amplification with complex nucleic acid from stool samples, full characterization may require metagenomic sequencing. However, our results from multiple molecular assays clearly demonstrated the presence of non-O1/O139 *V. cholerae* DNA in stools collected from nine asymptomatic Haitian infants two years prior to the earthquake.

## Figures and Tables

**Table 1:** Nucleotide sequences of PCR assays.

Target	Sequence (5’ - 3’)*	Detection	Reference
toxR	F: GTTTGGCGAGAGCAAGGTTT R: TCTCTTCTTCAACCGTTTCCA P: CGCAGAGTCGAAATGGCTTGG	TaqMan Probe	[5]
hlyA	F: ATCGTCAGTTTGGAGCCAGT R: TCGATGCGTTAAACACGAAG	SYBR Green with melting curve	[6]
O1-specific	F: CAACAGAATAGACTCAAGAA R: TATCTTCTGATACTTTTCTAC	SYBR Green with melting curve	[7]
O139-specific	F: TTACCAGTCTACATTGCC R: CGTTTCGGTAGTTTTTCTGG	SYBR Green with melting curve	[7]
ctxA	F:CGGGCAGATTCTAGACCTCCTG R:CGATGATCTTGGAGCATTCCCAC	SYBR Green with melting curve	[8]
ctxB	F: ACTATCTTCAGCATATGCACATGG R_classical_ CCTGGTACTTCTACTTGAAACG R_El tor_ CCTGGTACTTCTACTTGAAACA	SYBR Green with melting curve	[9]
zot	F:TCGCTTAACGATGGCGCGTTTT R:AACCCCGTTTCACTTCTACCCA	SYBR Green with melting curve	[8]
rtxA	F:CTGAATATGAGTGGGTGACTTACG R:GTGTATTGTTCGATATCCGCTACG	SYBR Green with melting curve	[10]
rtxC	F: CGACGAAGATCATTGACGAC R: CATCGTCGTTATGTGGTTGC	SYBR Green with melting curve	[10]

* F: forward primer; R: reverse primer; P: probe.
